# Acute Myeloid Leukaemia of Donor Cell Origin Developing 17 Years after Allogenic Hematopoietic Cell Transplantation for Acute Promyelocytic Leukaemia

**Published:** 2012-12

**Authors:** Pilar Jiménez, J. Carlos Alvarez, Pilar Garrido, J. Antonio Lorente, Jorge Palacios, Francisco Ruiz-Cabello

**Affiliations:** 1 *Servicio de Análisis Clínicos e Inmunología, Hospital Universitario Virgen de las Nieves, Granada, Spain;*; 2 *Departamento de Medicina Legal, Universidad de Granada, Spain;*; 3 *Servicio de Hematología, Hospital Universitario Virgen de las Nieves, Granada, Spain*

**Keywords:** bone marrow transplantation, donor cell leukaemia, FISH, MLL, STR

## Abstract

Donor cell leukaemia (DCL) is a rare complication of allogenic hematopoietic cell transplantation (HCT). We report the case of a female patient with acute promyelocytic leukaemia (APL), FAB type M3, who developed acute myeloid leukaemia (AML) type M5 of donor origin 17 years after allogenic bone marrow transplantation (BMT) from her HLA-matched sister. Morphology and immunophenotyping showed differences with the initial leukaemia, and short tandem repeat (STR) analysis confirmed donor-type haematopoiesis. Interphase fluorescence in situ hybridisation (FISH) showed an 11q23 deletion. Given that the latency period between transplant and development of leukaemia was the longest reported to date, we discuss the mechanisms underlying delayed leukaemia onset.

## INTRODUCTION

Allogenic hematopoietic cell transplantation (HCT) is a highly effective therapy for haematological malignancies but can be complicated by disease relapse or development of secondary solid or haematological malignancies. Most post-HCT leukaemias are relapses caused by regrowth of the original malignant cells and rarely result from oncogenic transformation of normal donor hematopoietic cells in the recipient, i.e., donor cell leukaemia (1, 2). Since its first description 30 years ago, most reported DCL cases have been acute myeloid leukaemia (3). Although the true incidence of DCL is difficult to establish, the European Group for Blood and Marrow Transplantation survey estimated a rate of 124 cases per 100,000 transplants and a latency period from transplantation to leukaemia onset of 17 months, ranging widely from of 4 to 164 months (4).

We report the case of a female patient with acute promyelocytic leukaemia, FAB type M3, who developed AML M5 of donor origin 17 years after allogenic BMT from her HLA-matched sister. Combined phenotype and genotype analyses verified the diagnosis of DCL.

## CASE REPORT

We report the case of a woman who was diagnosed with APL FAB type M3 at the age of 29 years in 1993. Initial induction chemotherapy with a 3 × 3 regimen (100 mg/m^2^ cytarabine and 60 mg/m^2^ daunorubicin) achieved complete remission and was followed by consolidation chemotherapy with a 7 × 3 regimen. Four months later, she underwent allogenic BMT from her HLA-identical sister after a conditioning regimen with total body irradiation (TBI) and cyclophosphamide (60 mg/Kg daily for two days). Cyclosporine and a short course of methotrexate were administered for prevention of graft versus host disease (GVHD), and successful engraftment without GVHD was achieved after the BMT.

The patient remained in complete haematological remission for 17 years. In March 2010, she was admitted to the hospital with respiratory symptoms. Peripheral blood analysis revealed an abnormal complete blood count, with haemoglobin of 12.4 g/dL [13-17 g/dL], white blood cell count of 124.8 × 10^9^/L [4-10 × 10^9^/L] and platelet count of 50 × 10^9^/L [150-410 × 10^9^/L]. A peripheral blood (PB) smear contained 85% myeloid blast cells. Flow cytometry showed the blast cells to be positive for CD33, CD15, CD64 and HLA DR, with a weak expression of CD13, CD11b and CD4; they were negative for CD14, intracytoplasmic myeloperoxidase (MPO) and T-cell and B-cell markers. AML FAB type M5 was diagnosed.

**Figure 1:**
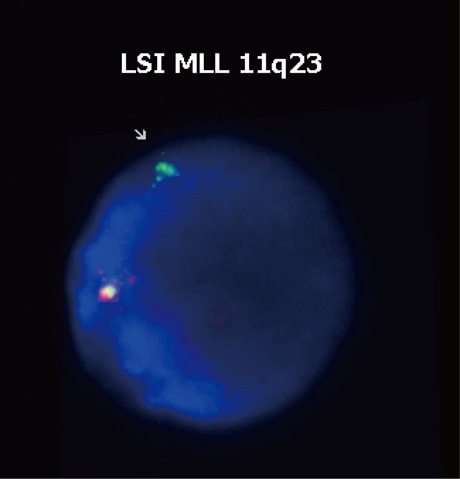
Dual-color fluorescence in situ hybridization (FISH) analysis of a bone marrow interphase with an *MLL break-apart* rearrangement probe. The figure depicts the detected pattern: one orange/green fusion signal corresponding to the normal chromosome 11q23 region and one isolated green signal. This pattern is characteristic of large deletions occurring distally from the *MLL* breakpoint that are detected in approximately 25% of 11q23 translocations.

**Figure 2:**
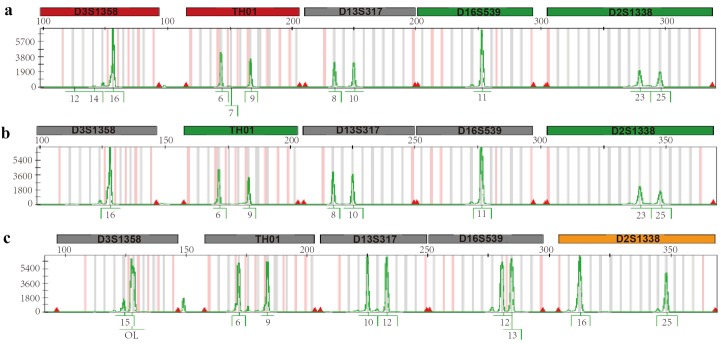
Molecular analysis of several STRs: electropherograms from (a) the donor´s PB cells; (b) the patient´s PB cells and (c) from the patient´s hair. D13S317, D16S539 and D2S1338 markers were able to discriminate the patient’s hair cells from the patient’s and donor’s PB cells. Signals in the patient’s and donor’s PB cells were identical and totally distinct from those in the DNA from the patient’s hair.

Cytogenetic analysis of bone marrow (BM) cells evidenced a normal female karyotype (46 XX). However, FISH on interphase nuclei using *MLL* break-apartrearrangementprobe showed a pattern of one orange/green fusion signal and one isolated green signal (Fig. 1); given that one orange signal was absent, the result can be interpreted as a 11q23 deletion occurring distally from the *MLL* breakpoint. Hence, the second AML did not exhibit the same phenotype as the original disease. The cell origin of the leukaemia was verified by analysing 23 short tandem-repeat markers. Because no molecular data were available for pre- and post-BMT chimerism monitoring, STR-PCR analysis was performed by comparing allelic patterns among PB from the patient at diagnosis, PB from the donor and DNA extracted from the patient’s hair (Fig. 2). STR-PCR analysis showed the same allele signals in PB samples from the patient and donor but different signals in samples from the patient´s hair, demonstrating the donor cell origin of the leukaemia.

The patient entered remission after induction chemotherapy (cytarabine and daunorubicin) in July 2010 and underwent only one consolidation course due to the onset of multiple complications, mainly respiratory. At the time of writing (June 2012), she is now disease–free, with normal BM morphologic, immunophenotyping and cytogenetic analysis results.

## METHODS

Immunophenotyping was performed by flow cytometry on freshly isolated BM cells. Cells were evaluated by four-colour analysis using a FACS Canto analyzer (Becton Dickinson, San José, CA, USA) with fluorescein isothiocyanate-, phycoerythrin-, PerCP-Cy5- and APC-conjugated monoclonal antibodies combined according to Euroflow antibody panels (5): anti-CD45, CD13, CD33, CD14, CD11b, CD64, CD36, CD34, CD15, CD19, CD20, CD3, CD4, CD8, CD10, CD56, CD38, CD117 and MPO (BD Biosciencies).

FISH analyses were performed on BM cells taken at AML relapse and prepared according to standard methods. Hybridization on interphase nuclei was achieved with the LSI MLL dual color, break apart rearrangement probe 11q23 (Vysis). The probe is designed to detect the 11q23 rearrangement associated with various translocations involving the MLL gene. The probe consists of a 350 kb portion centromeric to the MLL gene breakpoint cluster region (bcr) labelled in spectrum green and approximately a 190 kb portion largely telomeric of the bcr labelled in spectrum orange. A cell lacking the MLL rearrangement show a two orange/green (yellow) fusion signal pattern. In a cell possessing a MLL translocation, the expected pattern is one green/orange (yellow) fusion signal, one orange signal and one green signal. Slides were interpreted by scoring 200 interphase nuclei. Alteration was detected in 80% of the visualized nuclei.

STR analysis was performed as follows: genomic DNA was extracted from patient PB cells and donor PB cells and from the patient´s hair by standard methods, amplifying 23 STR loci by PCR. PCR products were analyzed by capillary electrophoresis (ABI Prism 310 Genetic Analyzer, Foster City, CA USA) using GeneScan Analysis 3.7 software.

## DISCUSSION

DCL is a rare complication of allogenic HCT (4). We present the case of a patient who developed a secondary AML at 17 years post-transplantation. Given its different phenotype from that of the first leukaemia and the very long latency period, we investigated its haematopoietic origin. Molecular analysis of 23 STR markers confirmed the donor cell origin of AML blasts.

The mechanism that produces leukaemia in previously healthy transplanted cells is not clear and may be distinct from that underlying other types of leukaemia (6). The pathogenesis of DCL is multifactorial, and intrinsic cell factors and factors in the host hematopoietic microenvironment have been implicated (7). Chronic tissue stress that can induce DNA damage and genomic alterations may result from chronic antigenic stimulation due to minor histocompatibility differences between donor and host cells and effects of chemotherapy and radiation therapy (8).

Several cytogenetic abnormalities have been reported in DCL (9-11). We found no chromosomal alterations in our patient by traditional cytogenetic methods but detected an 11q23 deletion by interphase analysis using a specific probe for the *MLL* region. AML with 11q23 abnormalities comprises one category of recurrent genetic abnormalities in the WHO classification. There are different possible causes of genetic *MLL* aberrations, including reciprocal chromosomal translocations, gene internal partial-tandem duplications and deletions and inversions of the long arm of chromosome 11 (12). These cytogenetically silent genomic alterations may be detectable by FISH and molecular analysis (13). The patient had received cytotoxic therapy with cytarabine and daunorubicin (topoisomerase II inhibitor) after the initial diagnosis. Topoisomerase II inhibitors are inducers of secondary leukaemia and are typically associated with 11q23 aberrations (11). However, some authors have suggested that *MLL* rearrangements are insufficient to confer cells with a selective proliferation/survival advantage (14); therefore, additional factors may be involved in the leukaemogenesis.

Post-HCT proliferative stress from the effort to repopulate the recipient´s bone marrow and the premature aging of donor cells also correlates with genomic instability and a higher likelihood of mutation (6). If a preleukaemic mutation occurs, a damaged bone marrow microenvironment and impaired immunosurveillance may favour emergence of a secondary neoplasm (4). HCT is often accompanied by clinical immunodeficiency in recipients, in which the following factors may play a role (11): recipients may be unable to regenerate a complete new lymphocyte repertoire; the graft may not contain a sufficient number and variety of lymphoid progenitors; in adult recipients, thymic involution may compromise proper T-cell maturation; and immunosuppressive drugs against GVHD can give rise to iatrogenic immunodeficiency due to T-cell abnormalities (7). Post-transplantation immune recovery takes months or years and may be incomplete in older patients (15). Delayed and incomplete recovery of T cells post-transplantation may create an imbalance between lymphoid and myeloid differentiation that favours the development of myeloid leukaemia (6). It has also been proposed that distinct mechanisms may underlie the early or late onset of DCL after HCT (8). The latency period (17 years) was longer in the present case than in previous reports of DCL. Failure to achieve complete restoration of the recipient immune defence mechanism may play an important role in late-onset DCL.

Transmission of occult leukaemic cells from donor to recipient has been reported as a very rare cause of DCL (9). However, the donor in this case did not develop haematological malignancies over the 17 years following the donation of hematopoietic cells.

The DCL and the initial leukaemia differed in morphologic, cytogenetic and immunophenotyping results. Accurate molecular diagnosis is essential to determine the donor origin of leukaemia. Cytogenetic and molecular characterization of DCL-associated genomic alterations may help to elucidate the mechanisms that underlie leukaemogenesis in order to prevent its development in transplant recipients.

## CONTRIBUTIONS TO THE WORK

J. Carlos Alvarez and J. Antonio Lorente performed and interpreted the STR analysis; P. Garrido and J. Palacios provided clinical data; F. Ruiz-Cabello performed immunophenotyping and revised the manuscript; P. Jiménez performed FISH analysis and wrote the paper.
